# A pharmacogenetic interaction analysis of bevacizumab with paclitaxel in advanced breast cancer patients

**DOI:** 10.1038/s41523-022-00400-6

**Published:** 2022-03-21

**Authors:** Luigi Coltelli, Giacomo Allegrini, Paola Orlandi, Chiara Finale, Andrea Fontana, Luna Chiara Masini, Marco Scalese, Giada Arrighi, Maria Teresa Barletta, Ermelinda De Maio, Marta Banchi, Elisabetta Fini, Patrizia Guidi, Giada Frenzilli, Sara Donati, Simona Giovannelli, Lucia Tanganelli, Barbara Salvadori, Lorenzo Livi, Icro Meattini, Ilaria Pazzagli, Marco Di Lieto, Mirco Pistelli, Virginia Casadei, Antonella Ferro, Samanta Cupini, Francesca Orlandi, Damiana Francesca, Giulia Lorenzini, Leonardo Barellini, Alfredo Falcone, Alessandro Cosimi, Guido Bocci

**Affiliations:** 1 Department of Oncology, Azienda USL Toscana Nord Ovest, Pisa, Italy; 2 Division of Medical Oncology, Livorno and Pontedera Hospitals, Azienda USL Toscana Nord Ovest, Pisa, Italy; 3 Department of Clinical and Experimental Medicine, University of Pisa, Pisa, Italy; 4 Division of Medical Oncology II, Azienda Ospedaliero-Universitaria Pisana, S. Chiara Hospital, Pisa, Italy; 5 Institute of Clinical Physiology, Italian National Research Council – CNR, Pisa, Italy; 6 Division of Medical Oncology, Versilia Hospital, Azienda Usl Toscana Nord Ovest, Lido di Camaiore, Italy; 7 Division of Medical Oncology, San Luca Hospital, Azienda Usl Toscana Nord Ovest, Lucca, Italy; 8 Division of Radiotherapy, Azienda Ospedaliero-Universitaria Careggi, Firenze, Italy; 9 Division of Medical Oncology, Pescia and Pistoia Hospitals, Azienda Usl Toscana Centro, Pistoia, Italy; 10 Division of Medical Oncology, Umberto I Salesi-Lancisi Hospital, Azienda Ospedaliero-Universitaria Umberto I, Ancona, Italy; 11 Division of Medical Oncology, Marche Nord Hospital, Azienda Ospedaliera San Salvatore, Pesaro, Italy; 12 Division of Medical Oncology, Santa Chiara Hospital, Azienda Provinciale per I Servizi Sanitari, Trento, Italy; 13 Division of Radiology, Pontedera Hospital, Azienda Usl Toscana Nord Ovest, Pisa, Italy; 14 Breast Unit – Division of Breast Surgery, Livorno Hospital, Azienda Usl Toscana Nord Ovest, Livorno, Italy; 15 Department of Translational Research and New Technology in Medicine and Surgery, University of Pisa, Pisa, Italy

**Keywords:** Breast cancer, Epistasis

## Abstract

To investigate pharmacogenetic interactions among *VEGF-A*, *VEGFR-2*, *IL-8*, *HIF-1α*, *EPAS-1*, and *TSP-1* SNPs and their role on progression-free survival (PFS) in metastatic breast cancer (MBC) patients treated with bevacizumab plus first-line paclitaxel or with paclitaxel alone. Analyses were performed on germline DNA, and SNPs were investigated by real-time PCR technique. The multifactor dimensionality reduction (MDR) methodology was applied to investigate the interaction between SNPs. The present study was an explorative, ambidirectional cohort study: 307 patients from 11 Oncology Units were evaluated retrospectively from 2009 to 2016, then followed prospectively (NCT01935102). Two hundred and fifteen patients were treated with paclitaxel and bevacizumab, whereas 92 patients with paclitaxel alone. In the bevacizumab plus paclitaxel group, the MDR software provided two pharmacogenetic interaction profiles consisting of the combination between specific *VEGF-A* rs833061 and *VEGFR-2* rs1870377 genotypes. Median PFS for favorable genetic profile was 16.8 vs. the 10.6 months of unfavorable genetic profile (*p* = 0.0011). Cox proportional hazards model showed an adjusted hazard ratio of 0.64 (95% CI, 0.5–0.9; *p* = 0.004). Median OS for the favorable genetic profile was 39.6 vs. 28 months of unfavorable genetic profile (*p* = 0.0103). Cox proportional hazards model revealed an adjusted hazard ratio of 0.71 (95% CI, 0.5–1.01; *p* = 0.058). In the 92 patients treated with paclitaxel alone, the results showed no effect of the favorable genetic profile, as compared to the unfavorable genetic profile, either on the PFS (*p* = 0.509) and on the OS (*p* = 0.732). The pharmacogenetic statistical interaction between *VEGF-A* rs833061 and *VEGFR-2* rs1870377 genotypes may identify a population of bevacizumab-treated patients with a better PFS.

## Introduction

The treatment of metastatic breast cancer (MBC) patients with hormone-receptors positive (HR+) and human epidermal receptor 2 negative (HER2−) tumors is dramatically changed over the years. In this setting, cyclin-dependent kinase 4/6 inhibitors (CDK4/6i), such as palbociclib, ribociclib and abemaciclib, in combination with aromatase inhibitors or fulvestrant represent today the first and later lines of therapy^1^.

However, chemotherapy-based treatment is still a therapeutic choice when hormone resistance occurs, in triple-negative tumor or in case of visceral crisis^2,3^. In this scenario, the humanized monoclonal antibody bevacizumab, in combination with paclitaxel, is a treatment option compared to chemotherapy alone^4^. Although a significant improvement in progression-free survival (PFS) was observed from three comparative studies, the US Food and Drug Administration (FDA), but not the European Medicines Agency (EMA), revoked the initial approval of bevacizumab for the first-line treatment of MBC patients because of the lack of benefit in terms of overall survival (OS). However, it has been theorized that when a long survival post first-line progression is expected after a first-line chemotherapy, such as in breast cancer, the lack of an apparent benefit in OS could not mean a lack of improvement in OS for the first line of treatment^4–8^.

Different strategies have been investigated to find possible predictive biomarkers and select those patients with the best chance of response to bevacizumab. Indeed, the PFS improvement due to bevacizumab was identical for magnitude in all subgroups of patients with different clinical and pathological characteristics^9^, and therefore new selective biomarkers should be needed to identify those patients who can have a major advantage in terms of outcome. Despite many attempts have been done, no validated biomarkers are currently available in the clinical practice and the prospective MERiDiAN trial failed to demonstrate a possible role of VEGF-A baseline in predicting the response to bevacizumab in breast cancer patients^10–15^.

Germline and somatic polymorphisms of genes involved in the angiogenic pathways have also been widely investigated in this research area to predict bevacizumab outcome, with contrasting results^12,16–18^. Due to the retrospective nature of these studies and to their inconclusive results, the role of single nucleotide polymorphisms (SNPs) as predictive markers remains to define^19^. Therefore, the current approach of correlating the bevacizumab response to a single SNP may be replaced by a genetic analysis of the interaction between SNPs, defined as non-linear interaction or epistasis. Moore and colleagues have established and validated a methodology, called multifactor dimensionality reduction (MDR) analysis, to identify a genetic profile with the ability to predict the drug response^20^. To test this hypothesis, we conducted a retrospective study on 113 MBC patients to assess the ability of MDR methodology to identify a favorable pharmacogenetic profile associated to PFS in patients treated with bevacizumab, combined with first-line paclitaxel. The MDR analysis provided two pharmacogenetic interaction profiles consisting of the combination between specific *VEGFR-2* rs11133360 and *IL-8* rs4073 genotypes. The median PFS was 14.1 months (95% CI, 11.4–16.8) and 10.2 months (95% CI, 8.8–11.5) for the favorable and the unfavorable genetic profile, respectively (HR = 0.44; 95% CI, 0.29–0.66; *p* < 0.0001)^21^.

Based on these encouraging results, our study was planned to evaluate the effects of the combination of paclitaxel with bevacizumab on patients harboring other different genetic profiles, exploring the possibility to predict the best favorable profile in terms of PFS, our primary endpoint. The second step was to test if the eventual seen PFS advantage could be maintained also in terms of OS (our secondary endpoint) in these patients even after the end of the administration of bevacizumab combined therapy. The analysis was extended to a group of patients treated without bevacizumab, during the same period of time, with the purpose of having a control group.

## Results

### Patients

Two-hundred and fifteen patients treated with bevacizumab in combination with paclitaxel and 92 patients treated with first-line chemotherapy without bevacizumab, entered the present MDR analysis. In the bevacizumab plus paclitaxel group, the median number of cycles administered were 7 (range 4–18) and maintenance with bevacizumab alone was continued in 152 patients (70.7%).

All the 215 patients were evaluated for the response. 21 (10%) and 126 (58%) experienced a complete and a partial response, respectively; 52 patients (24%) reported a stable disease (SD) and in 16 patients (8%) a progression was observed. None of the analyzed polymorphisms was associated with the response rate (data not shown).

When the present analysis was performed, 215 out of 215 patients (100%) progressed and 170 out of 215 patients (79%) died from the metastatic disease. No patients died of cancer-unrelated causes. The median PFS and median OS were 11.8 months (95% CI, 10.9–12.7 months) and 30.7 months (95% CI, 26–35.5 months), respectively. Data were censored after 70 months.

The main characteristics of the patients are reported in Table 1. While the differences observed between groups may be due to the retrospective nature of the study, the characteristics between favorable and unfavorable group were superimposable.

**Table 1:** Characteristics of patients at baseline treated with paclitaxel (PTX) plus bevacizumab (BEV) or paclitaxel alone.

		PTX + BEV	% PTX + BEV	PTX	% PTX	*p*-value
Adjuvant CHT	No	76	35.3	42	45.7	0.089
	Yes	139	64.7	50	54.3	
Adjuvant CHT with taxanes	No	157	73.4	71	77.2	0.483
	Yes	57	26.6	21	22.8	
Adjuvant HT	No	84	39.4	35	38	0.819
	Yes	129	60.6	57	62	
DFI ≥ 12 months	No	57	26.5	31	33.7	0.202
	Yes	158	73.5	61	66.3	
≥3 Pathological sites	No	145	67.8	74	80.4	0.024
	Yes	69	32.2	18	19.6	
Visceral disease	No	68	31.6	38	41.3	0.102
	Yes	147	68.4	54	58.7	
HR positive	No	37	17.5	9	9.8	0.086
	Yes	175	82.5	83	90.2	
Age > or <65 years	>	171	79.5	48	52.2	0.0001
	<	44	20.5	44	47.8	

*CHT* chemotherapy, *DFI* disease-free interval, *HR* hormone receptor, *HT* hormonal therapy.

### Association of clinical and pathological characteristics with PFS and OS

In Table 2 are reported the associations of clinical and pathological characteristics with both PFS and OS in the 215 patients treated with paclitaxel and bevacizumab. Hormonal-receptor status confirmed its role in determining the prognosis of this group of patients. Interestingly, in the group of patients who continued bevacizumab, over the patients who interrupted it at the end of chemotherapy without evidence of a disease progression, both a greater PFS and OS was observed. In Table 3 are reported the associations of clinical and pathological characteristics with PFS in the group of patients (*n* = 92 that received paclitaxel without bevacizumab).

**Table 2:** Association between clinical and pathological characteristics with progression-free survival (PFS) and overall survival (OS) in 215 patients treated with paclitaxel and bevacizumab.

Characteristics		*N* (215)	PFS			OS		
				95% CI	P	HR	95% CI	*p*
ECOG PS	0	190			0.124			0.004
	1	23	1.495	0.965–2.316	0.072	2.053	1.281–3.291	0.003
	2	2	2.094	0.517–8.478	0.300	4.544	0.625–33.033	0.135
Hormonal receptor	Negative	37						
	Positive	175	0.501	0.349–0.721	0.0001	0.470	0.318–0.694	0.0001
DFI	<12 mos	30						
	≥12 mos	83	1.045	0.767–1.425	0.779	0.910	0.651–1.272	0.581
Sites involvement	<3	145						
	≥3	69	1.361	1.016–1.823	0.039	1.336	0.972–1.838	0.075
Adjuvant CHT	Yes	139	1.240	0.932–1.649	0.140	1.124	0.820–1.540	0.467
	No	76						
Adjuvant taxanes	Yes	57	1.059	0.778–1.443	0.715	1.131	0.800–1.599	0.0487
	No	157						
Visceral disease	Yes	147	1.108	0.827–1.484	0.494	1.256	0.904–1.745	0.175
	No	68						

*CHT* chemotherapy, *DFI* disease-free interval, *ECOG PS* Eastern Cooperative Oncology Group performance status, *HR* hazard ratio, *mos* months.

**Table 3:** Association between clinical and pathological characteristics with progression free survival (PFS) in 92 patients treated with paclitaxel without bevacizumab.

Characteristics		*N* (92)	PFS		
			HR	95% CI	*p*
ECOG PS	0	65	0.657		0.0015
	1	19	0.506	0.381–1.608	0.82
	2	5	0.587	0.475–3.730	0.41
	NA	3	–	–	–
Hormonal receptor	Negative	9			
	Positive	83	1.326	0.635–2.771	0.453
DFI	<12 months	31			
	≥12 months	61	1.534	0.980–2.401	0.061
Sites involvement	<3	74			
	≥3	18	1.376	0.806–2.350	0.242
Adjuvant CHT	Yes	50	1.569	1.001–2.457	0.049
	No	42			
Adjuvant taxanes	Yes	21	2.138	0.283–3.562	0.004
	No	71			
Visceral disease	Yes	54	2.036	1.289–3.215	0.002
	No	38			

*CHT* chemotherapy, *DFI* disease-free interval, *HR* hazard ratio, *NA* not assessable.

The Cox proportional hazards analysis was applied to evaluate the association between each single polymorphism with both PFS and OS. The analysis did not reveal significant positive association between each SNP with PFS (Table 4). No significant associations were observed with OS (data not shown).

**Table 4:** Association between each polymorphism and progression free survival in patients treated with paclitaxel and bevacizumab.

Polymorphisms	Genes	Carriers	*N*	HR	95% CI	*p*
rs699947	*VEGF-A*	AA	37	1		
		AC	110	1.054	0.708–1.570	0.796
		CC	68	1.016	0.658–1.567	0.943
rs833061	*VEGF-A*	CC	36	1		
		CT	111	1.051	0.702–1.572	0.810
		TT	68	1.069	0.689–1.659	0.766
rs3025039	*VEGF-A*	CC	156	1		
		CT	50	0.927	0.667–1.289	0.653
		TT	9	0.500	0.248–1.009	0.053
rs1570360	*VEGF-A*	GG	45	1		
		AG	87	1.041	0.716–1.513	0.833
		AA	83	1.550	1.063–2.259	0.023
rs699946	*VEGF-A*	AA	123	1		
		AG	83	0.773	0.573–1.041	0.090
		GG	9	0.872	0.399–1.904	0.731
rs2010963	*VEGF-A*	GG	82	1		
		CG	109	0.921	0.681–1.246	0.595
		CC	24	0.775	0.471–1.276	0.317
rs2305948	*VEGFR-2*	CC	177	1		
		CT	35	1.169	0.801–1.708	0.418
		TT	3	2.722	0.854–8.672	0.090
rs11133360	*VEGFR-2*	TT	64	1		
		CT	109	0.947	0.682–1.315	0.745
		CC	42	1.048	0.695–1.578	0.824
rs2071559	*VEGFR-2*	AA	57	1		
		AG	107	1.182	0.843–1.657	0.332
		GG	51	1.336	0.906–1.969	0.144
rs1870377	*VEGFR-2*	TT	125	1		
		AT	78	0.983	0.727–1.330	0.912
		AA	12	0.938	0.514–1.712	0.836
rs11549465	*HIF-1α*	CC	44	1		
		CT	164	1.180	0.820–1.699	0.373
		TT	7	1.153	0.500–2.656	0.739
rs4145836	*EPAS-1*	GG	158	1		
		AG	53	1.063	0.765–1.477	0.716
		AA	4	0.504	0.185–1.371	0.179
rs4073	*IL-8*	AA	39	1		
		AT	114	0.952	0.647–1.400	0.802
		TT	62	0.782	0.510–1.198	0.258

*CI* confidence interval, *EPAS-1* endothelial PAS domain-containing protein 1, *HIF-1α* hypoxia-inducible factor-1α, *HR* hazard ratio, *IL-8* interleukin-8, *TSP-1* thrombospondin-1, *VEGF-A* vascular endothelial growth factor-A, *VEGFR-2* VEGF receptor-2.

Hormonal receptor status, bevacizumab maintenance and number of sites involvement are the covariates used for the Cox proportional hazards analysis. A *p* value < 0.00357 was defined as statistically significant (Bonferroni’s correction).

### MDR analysis

The MDR analysis revealed a genetic interaction profile, consisting of the combination between specific *VEGF-A* rs833061 and *VEGFR-2* rs1870377 genotypes, significantly associated with PFS and OS benefit. Particularly, two pharmacogenetic profiles were identified in patients, as reported in Table 5. The first one was associated with a greater PFS and OS benefit, whereas the second one with a lower PFS and OS after paclitaxel plus bevacizumab treatment. The characteristics at baseline of patients treated with paclitaxel alone or paclitaxel + bevacizumab harboring the pharmacogenetic favorable and unfavorable profile are reported in Tables 6 and 7, respectively.

**Table 5:** Results of the genetic interaction analysis to translate the genotype combinations of the *VEGF* rs833061 and *VEGFR-2* rs1870377 polymorphisms into favorable or unfavorable genetic profiles for progression-free survival.

*Favorable genetic profiles*			
*VEGF-A* rs833061	CC	*VEGFR-2* rs1870377	TT
*VEGF-A* rs833061	CT	*VEGFR-2* rs1870377	AT
*Unfavorable genetic profiles*			
*VEGF-A* rs833061	TT	*VEGFR-2* rs1870377	TT
*VEGF-A* rs833061	TT	*VEGFR-2* rs1870377	AT
*VEGF-A* rs833061	TT	*VEGFR-2* rs1870377	AA
*VEGF-A* rs833061	CT	*VEGFR-2* rs1870377	TT
*VEGF-A* rs833061	CT	*VEGFR-2* rs1870377	AA
*VEGF-A* rs833061	CC	*VEGFR-2* rs1870377	AT
*VEGF-A* rs833061	CC	*VEGFR-2* rs1870377	AA

*VEGF-A*, vascular endothelial growth factor A; *VEGFR-2*, vascular endothelial growth factor receptor-2.

**Table 6:** Characteristics of patients treated with paclitaxel alone with pharmacogenetic favorable (fav) and unfavorable (unfav) profile at baseline.

		fav (N)	% *N*	unfav (*N*)	% *N*	*p*-value
Adjuvant CHT	No	11	50.0	31	44.3	0.639
	Yes	11	50.0	39	55.7	
Adjuvant CHT with taxanes	No	14	63.6	57	81.4	0.083
	Yes	8	36.4	13	18.6	
Adjuvant HT	No	6	27.3	29	41.4	0.233
	Yes	16	72.7	41	58.6	
DFI ≥ 12 months	No	7	31.8	24	34.3	0.831
	Yes	15	68.2	46	65.7	
≥3 Pathological sites	No	17	77.3	57	81.4	0.668
	Yes	5	22.7	13	18.6	
Visceral Disease	No	9	40.9	29	41.4	0.966
	Yes	13	59.1	41	58.6	
HR positive	No	0	0.0	9	12.9	0.077
	Yes	22	100.0	61	87.1	
Age > or <65 years	>	12	54.5	36	51.4	0.799
	<	10	45.5	34	48.6	

*CHT* chemotherapy, *DFI* disease-free interval, *HR* hormone receptor, *HT* hormonal therapy.

**Table 7:** Characteristics of patients treated with paclitaxel plus bevacizumab with pharmacogenetic favorable (fav) and unfavorable (unfav) profile at baseline.

		fav (*N*)	% *N*	unfav (N)	% *N*	*p*-value
Adjuvant CHT	No	24	36.4	52	34.9	0.836
	Yes	42	63.6	97	65.1	
Adjuvant CHT with taxanes	No	49	74.2	108	73.0	0.846
	Yes	17	25.8	40	27.0	
Adjuvant HT	No	29	44.6	55	37.2	0.305
	Yes	36	55.4	93	62.8	
DFI ≥ 12 months	No	20	30.3	37	24.8	0.402
	Yes	46	69.7	112	75.2	
≥3Pathological sites	No	49	74.2	96	64.9	0.175
	Yes	17	25.8	52	35.1	
Visceral disease	No	22	33.3	46	30.9	0.720
	Yes	44	66.7	103	69.1	
HR positive	No	10	15.2	27	18.5	0.553
	Yes	56	84.8	119	81.5	
Age > or <65 years	>	10	84.8	34	22.8	0.199
	<	56	15.2	115	77.2	

*CHT* chemotherapy, *DFI* disease-free interval, *HR* hormone receptor, *HT* hormonal therapy.

**Fig. 1:**
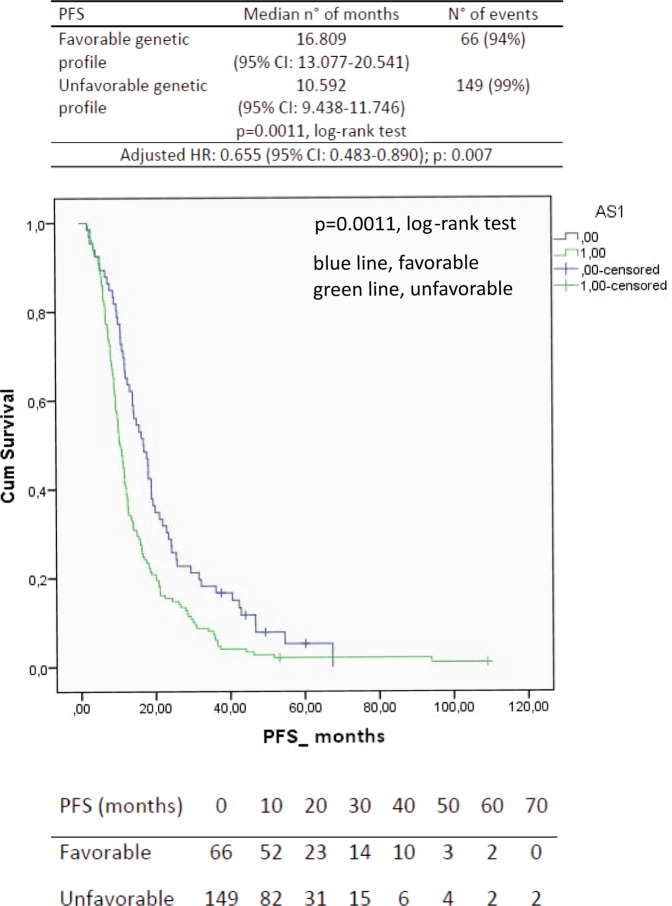
Progression-free survival (PFS) curves in patients treated with paclitaxel and bevacizumab calculated by the Kaplan–Meier method, according to the favorable (blue line) and unfavorable (green line) genetic profiles, with the adjusted hazard ratio (HR). CI confidence interval.

**Fig. 2:**
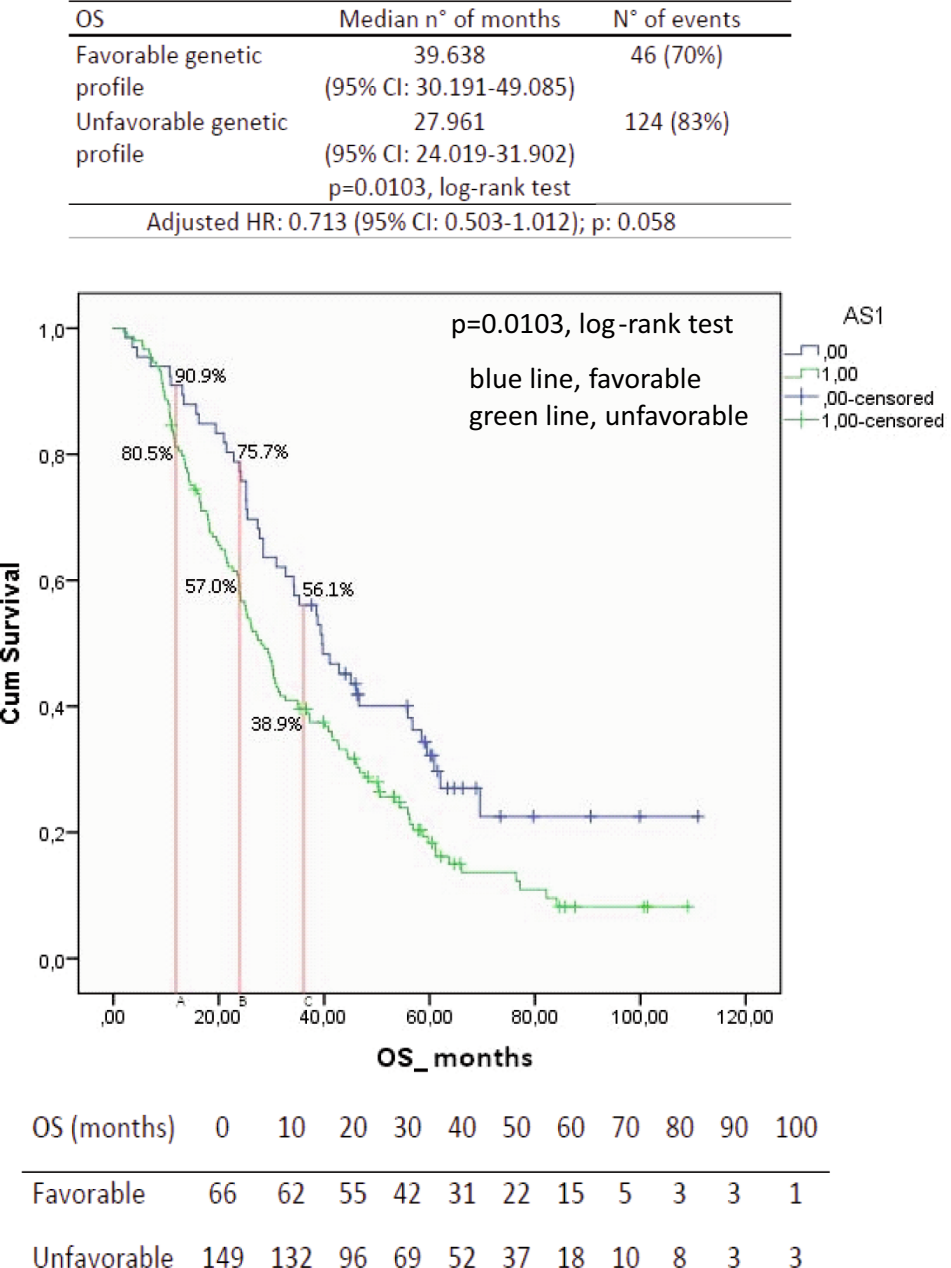
Overall survival (OS) curves in patients treated with paclitaxel and bevacizumab calculated by the Kaplan–Meier method, according to the favorable (blue line) and unfavorable (green line) genetic profiles, with the adjusted hazard ratio (HR). CI confidence interval.

The median PFS for the favorable genetic profile was 16.8 months (95% CI, 13.1–20.5 months) vs. the 10.6 months of the unfavorable genetic profile (95% CI, 9.4–11.7 months; *p* = 0.0011, log-rank test; Fig. 1). The Cox proportional hazards model, which was performed to assess the adjusted hazard ratio for the PFS of the favorable genetic profile, showed a value of 0.64 (95% CI, 0.5–0.9; *p* = 0.004; Table 8). Furthermore, a formal test of interaction confirmed the predictive nature of the favorable profile in the bevacizumab + paclitaxel group as reported in supplementary Table 1. Remarkably, the patients included in the favorable genetic profile also had the best probability of OS benefit, and the difference was significant as compared to the OS of the unfavorable genetic profile (Fig. 2). The median OS for the favorable genetic profile was 39.6 months (95% CI, 30.2–40.1 months) vs. the 28 months of the unfavorable genetic profile (95% CI, 24–32 months; *p* = 0.0103, log-rank test; Fig. 2). The Cox proportional hazards model, including the same significant parameters described in Table 8, revealed an adjusted hazard ratio for the OS of the favorable genetic profile of 0.71 (95% CI, 0.5–1.01; *p* = 0.058), at the limit of significance. Of note, the probability of an estimated 1-year survival rate was 90.9% (95% CI, 90.4–91.4) in the favorable genetic profile and 80.5% (95% CI, 80.2–80.8) in the unfavorable genetic profile; the estimated 2-year survival was 75.7% (95% CI, 75.2–76.2) and 57% (95% CI, 56–57.4), respectively. The estimated 3-year survival rate was 56.1% (95% Cl, 56.1–57.1) in the favorable genetic profile and 38.9% (95% Cl, 38.4–39.3) in the unfavorable. The observed objective responses were 69.7% in the favorable genetic profile as compared with 69.1% in the unfavorable genetic profile.

**Table 8:** Multivariable Cox regression model, including significant variables at the univariate analysis in patients treated with paclitaxel and bevacizumab.

Progression-free survival (*N* = 215)				
Characteristics		HR	95% CI	*p*
Hormone Receptor	Negative	1		
	Positive	0.48	0.33–0.70	<0.0001
Sites involvement	<3	1		
	≥3	1.51	1.11–2.04	0.008
Favorable genetic profile	No	0.64	0.47–0.86	0.004
	Yes	1		

*HR* hazard ratio, *CI* confidence interval

Also, the 92 MBC patients treated with a first-line chemotherapy including paclitaxel without bevacizumab were investigated to test the impact of the two genetic profiles in both PFS and OS. The results revealed no effect of the favorable genetic profile, as compared to the unfavorable genetic profile, either on the PFS (*p* = 0.509, log-rank test; Supplementary Fig. 1a) or on the OS (*p* = 0.732, log-rank test; Supplementary Fig. 1b).

## Discussion

The current standard therapy of patients suffering of metastatic breast cancer with HR+ and HER2− disease, in first and later lines of treatment, is represented by combinations of hormone and novel targeted therapies. The CDK4/6i palbociclib, ribociclib, and abemaciclib in combination with aromatase inhibitors or fulvestrant have dramatically changed the treatment of this setting of patients^1^.

Despite these treatments’ advances, the chemotherapy maintains its key role because almost all metastatic patients with HR+ and HER2− disease develop resistance over time to endocrine therapy. As well, chemotherapy represents the first choice of treatment in triple negative, *BRCA* wild type and PD-L1 negative disease^3^.

In this *scenario*, bevacizumab in combination with paclitaxel can still represent an option but the lack of any advantage in terms of OS, led to a slow decline in its use in the clinical practice during last years. Moreover, a recent meta-analysis has investigated, in head-to-head comparison, the role of endocrine treatment *versus* chemotherapy in postmenopausal setting with HR+ and HER2− metastatic disease, highlighting that bevacizumab in combination with paclitaxel was the only regimen that was significantly better than palbociclib plus letrozole in terms of response rate^22^. Thus, the identification of pharmacodynamic biomarkers could better select patients with the best chance of bevacizumab response and clarify the role of this antiangiogenic antibody in the management of MBC patients.

The multifactor dimensionality reduction (MDR) methodology has been previously used to identify genetic polymorphisms interactions profiles able in predicting drug response in metastatic colorectal cancer patients. In the study published by Pander and colleagues^23^, an interaction between VEGF+ 405G>C and TYMS-TSER polymorphisms, instead of an individual polymorphism, seemed to predict the CAPOX-B (capecitabine, oxaliplatin, and bevacizumab combination) response in terms of PFS, suggesting a paradigm shift from SNPs to a more complex interaction gene analysis able to predict response to antitumor agents.

The current study was planned to evaluate the effects of the combination of paclitaxel with bevacizumab *versus* paclitaxel alone on MBC patients harboring different genetic profiles, exploring the possibility to predict the best favorable profile in terms of PFS. The second step was to test if the eventual seen PFS advantage could be maintained also in terms of OS in these patients. The previous study on *VEGFR-2* and *IL-8* genetic interaction analysis^21^, the favorable profile in terms of PFS was not predictive of OS benefit. In the present study, the seen advantage in PFS was indeed confirmed in OS (an adjusted hazard ratio of 0.71) but with a *p* = 0.058, a value very close to a statistically significance, but not significant. However, the reported data, although statistically negative, seem to suggest that the favorable profile in terms of PFS may probably be maintained also in terms of OS and undoubtedly merits further investigations in a validation prospective study. Indeed, evaluation and confirmation of these findings in an independent cohort is critical because of the exploratory nature of our ambidirectional trial.

The analyses conducted with the MDR methodology in this unselected population of MBC patients revealed more than a genetic interaction profile, consisting of the combination between specific genotypes, but, due to nature of the MDR methodology, we investigated the genetic profile with a benefit in terms of both PFS and OS. The analysis conducted revealed a genetic interaction profile, consisting of the combination between specific genotypes of *VEGF* rs833061 and *VEGFR-2* rs1870377. Particularly, two genetic profiles were identified in patients, as reported in Table 5. The first one was associated with a greater both PFS and OS benefit compared to the second one. However, this model considered all the candidates and allows for any and all combination of SNPs to correlate with outcome. Thus, there are other significant or borderline permutations. Indeed, we have also included, as an example in the supplementary data (Supplementary Table 2 and Supplementary Fig. 2), another interesting genetic profile with a significant advantage in term of PFS but without any advantage in OS (not even a tendency).

Therefore, in our study we demonstrated, through the MDR methodology, a statistical interaction between *VEGF-A* and *VEGFR-2* gene SNPs that potentially relates to bevacizumab efficacy on both PFS and OS. The two genes, and, consequently, the two proteins belong to the same signaling pathway, and it has been clearly demonstrated that VEGF-A stimulates VEGFR-2 phosphorylation and tumor angiogenesis^24^. Based on these premises, it is conceivable to hypothesize that, in patients carrying the favorable genetic profile, the tumor angiogenesis is successfully inhibited in the presence of bevacizumab. The pharmacological inhibition of the angiogenic process by bevacizumab could be effective because of the physiological (not increased) production of VEGF-A due to the presence of *VEGF-A* rs833061 CC genotype or C allele. Indeed, for this SNP *VEGF-A* rs833061 C>T it has been described an increased promoter activity due to the T allele^25^ that may explain an eventual resistance to the treatment. Moreover, the *VEGFR-2* rs1870377 is a nonsynonymous SNP substituting glycine with histidine (Q472H) located in the extracellular ligand binding region of the receptor, potentially impacting VEGFR-2 degradation^26^. The *VEGFR-2* rs1870377 TT genotype or T allele present in the favorable profile synthetize a receptor not modified in its structure, suggesting that it is not abnormally activated or degraded. Therefore, it might be plausible that the genetic background characterized by a physiological activation of the VEGF-A pathway may be responsible, in part, for the positive effect of bevacizumab maintenance therapy in these MBC patients. In contrast, in patients with an unfavorable genetic profile, the microenvironment conditions due to the different genotype combinations may result in an increase of the VEGF-A production and/or the presence of an altered VEGFR-2 on tumor endothelial cells which may be capable to proliferate, migrate or survive because the VEGF action is not completely blocked by bevacizumab.

The absence of any advantage in terms of efficacy in the patients treated with chemotherapy without bevacizumab could suggest a possible predictive role of the favorable genetic profile for bevacizumab response, but the exploratory nature of this ambidirectional study may limit this hypothesis. However, the main findings of our analyses support the conclusion that a genetic profile may identify a group of patients with longer PFS and OS, predicting the response to bevacizumab in combination with paclitaxel.

The MDR approach is a major reason for differences between our trial and other studies on bevacizumab biomarkers such as E2100^16^. There are additional aspects between the E2100 US patients and the Italian population of our study that may account for different results. First of all, Italian patients were of Caucasian origin and no patients of African origin were represented. Secondly, although the frequencies of our studied *VEGF-A* and *VEGFR-2* SNPs were superimposable with the ones of the Caucasian patients published in the article by Schneider and colleagues^16^, there was an exception regarding the VEGFR-2 889A/G (rs2071559). In that case, the frequency of the minor allele A in our population was 0.49 whereas in the E2100 study was 0.09.

New pharmacogenetic favorable biomarkers of bevacizumab-combined therapies could be retrieved from a genetic analysis of the interaction among SNPs rather than from the examination of a single SNP of a single gene. Surely, a multigene-risk biomarkers may be more beneficial from a comprehensive agnostic approach using genome-wide association studies (GWAS) rather than a candidate gene approach as the one that we have used in our study. However, some challenges have been faced when scientists tried the scaling of MDR to big data, as the one from GWAS, such as the necessity to filter the data prior to MDR analysis^27^, also using biological knowledge through tools such as BioFilter^28^. Moreover, our work can definitively be strengthened by the biological characterization of the VEGF expression in the pre-treatment tissue. Indeed, since rs833061 is located in the promoter region of VEGF-A, the difference in expression levels of VEGF-A in tumors of patients harboring the favorable vs. unfavorable profile could be an important strategy to confirm our statistical findings.

In conclusion, the MDR methodology could be successfully used as witnessed by the experience in this unselected MBC patients where the investigation of an interaction between *VEGF-A rs833061 and VEGFR-2 rs1870377* gene polymorphisms resulted in the identification of a genetic profile associated with a longer PFS.

## Methods

### Study population

This is an explorative, ambidirectional cohort study, meaning that eligible patients were enrolled and evaluated retrospectively from January 2009 until September 2016 and then followed prospectively. The oncology units, all located in the north or center of Italy, were selected based on their clinical experience in the use of the combination of paclitaxel and bevacizumab as first-line therapy in histologically confirmed HER-2-negative MBC patients. Two-hundred fifteen patients from 11 Italian divisions of Medical Oncology, with histologically confirmed HER2-negative MBC, were treated with a first-line therapy including bevacizumab 10 mg/m^2^ i.v. on days 1 and 15 combined with first-line paclitaxel 90 mg/m^2^ i.v. on days 1, 8, and 15, every 4 weeks, and they were enrolled for the present pharmacogenetic study. Ninety-two MBC patients treated with a first-line chemotherapy including paclitaxel without bevacizumab, during the same period, were also included into the study as a control group. The patients enrolled in the previously published study^21^ have been also included in the present analysis. Basal and pathological characteristics recorded from both groups were the following: age (≤ or >65 years); Eastern Cooperative Oncology Group (ECOG) performance status (0 or 1–2); hormonal-receptor status (positive or negative); previous adjuvant chemotherapy (none, anthracycline or anthracycline plus taxanes); previous hormonal therapy (adjuvant or metastatic); disease-free interval from the first diagnosis of breast cancer (≤ or >12 months); extent of disease (≤ or >3 sites); location of disease (viscera or bone); disease evaluation (measurable or non-measurable). Patients with human epidermal growth factor receptor type 2 (HER2)-positive, were excluded from the present study. The characteristics of the patients are summarized in Table 1.

The treatment with chemotherapy was continued until either disease progression occurred or unacceptable toxicities registered, or it was stopped for medical choice. The bevacizumab maintenance was continued, and hormone therapy added for both groups when indicated, until disease progression or unacceptable toxicities occurred.

Sites of metastatic disease were radiologically re-evaluated according to the RECIST criteria 1.1, in patients with measurable disease, every 2 months. In patients without measurable lesions, progression of disease was defined when new lesions appeared or when existing lesions evolved. Likewise, in the case of non-measurable lesions, deterioration of clinical condition not due to treatment toxicity, was defined as progression of disease.

PFS was defined as the period from the beginning of the treatment to the first observation of disease progression as above described, or death from any cause. OS was defined as the period from the beginning of the treatment to death from any cause. All patients were assessed for response, PFS and OS. Each patient entering the study signed the informed consent. The disease assessment was conducted by the investigators based on the approved protocol and all the oncology units followed the same assessment schedule and criteria for the prospective follow-up. The protocol was approved by ethic committee of Azienda Ospedaliera-Universitaria Pisana (CESM-AOUP 3077/2010; clinicaltrials.gov identifier NCT01935102) for Pisa, Livorno, Lucca, Massa Carrara, Versilia, and Pontedera Hospitals, and by the ethic committees of all participating centers.

### Genotyping analyses

Blood samples (3 ml) were collected in EDTA tubes and stored at −80 °C. Genes and polymorphisms, involved in the angiogenesis pathways, were selected for the present analyses based on our previous study^21^. In the Table 9, the selected polymorphisms are reported. Germline DNA extraction was performed using QIAamp DNA Blood Mini Kit (Qiagen, Valencia, CA, USA). Allelic discrimination of genes was performed using an ABI PRISM 7900 SDS (Applied Biosystems, Carlsbad, CA, USA) and with validated TaqMan^®^ SNP genotyping assays (Table 9; Applied Biosystems). PCR reactions were carried out according to the manufacturer’s protocol. Genotyping was not performed until an adequate number of events (>80% on study population) was reported in terms of PFS. All the samples were analyzed twice to replicate the obtained genotype.

**Table 9:** Selected genes and polymorphisms of the present pharmacogenetic study.

Gene	rs number	TaqMan SNP genotyping assays
*VEGF-A*		
	rs699947	C__8311602_10
	rs833061	C__1647381_10
	rs3025039	C__16198794_10
	rs1570360	C__1647379_10
	rs699946	C__1647395_10
	rs2010963	C__8311614_10
*VEGFR-2*		
	rs2305948	C__22271999_20
	rs11133360	C__26111278_10
	rs2071559	C__15869271_10
	rs1870377	C__11895315_20
*HIF-1α*		
	rs11549465	C__25473074_10
*TSP-1*		
	rs2228262	C__16170900
*EPAS-1*		
	rs4145836	C__32329435_10
*IL-8*		
	rs4073	C__11748116_10

*EPAS-1* endothelial PAS domain-containing protein 1 (also known as hypoxia-inducible factor-2α), *HIF-1α* hypoxia-inducible factor-1α, *IL-8* interleukin-8, *TSP-1* thrombospondin-1, *VEGF-A* vascular endothelial growth factor-A, *VEGFR-2* VEGF receptor-2.

### Statistical analysis

The investigators responsible for data analysis were blinded to which samples were from patients treated with paclitaxel alone and paclitaxel plus bevacizumab.

The aim of the present study was to identify a favorable genetic profile in terms of PFS in MBC patients treated with bevacizumab in association with paclitaxel. The corresponding OS in these patients remained a secondary endpoint as well as response rate. All polymorphisms were analysed for deviation from the Hardy–Weinberg Equilibrium (HWE) by means of comparison between observed allelic distributions with those expected from the HWE by on *χ*^2^ test (see Supplementary Tables 3 and 4).

Any association between gene polymorphisms and response rate was analysed by the two-sided Fisher’s exact test. The association between each individual polymorphism and the most relevant clinical-pathological characteristics with PFS and OS was tested using a Cox proportional hazards model. In these analyses we used Bonferroni’s correction and the *p* value <0.00357 (0.05/14 SNPs = 0.00357) was accepted as statistically significant. The multifactor dimensionality reduction (MDR) methodology was applied (MDR software version 2.0 beta 6 on http://sourceforge.net/projects/mdr/, last access January 2021) to investigate the interaction between gene polymorphisms and to identify favorable genetic profiles associated with the greater PFS in this population of patients. MDR was developed as a non-parametric and genetic model-free data mining strategy for identifying combinations of SNPs that are predictive of a discrete clinical endpoint. MDR approach is a constructive induction algorithm that creates a new attribute by pooling genotypes from multiple SNPs^29,30^. The difference both in PFS and OS between favorable genetic profiles and the unfavorable genetic profiles were assessed with the log-rank test and the Kaplan–Meier method to evaluate survival curves. A Cox proportional hazards model, with the possible genetic profiles and the clinical and pathological patient’s characteristics individually related with both the PFS and OS, was used to calculate the adjusted hazards ratio (HR) and the 95% confidence interval (95% CI). The Kaplan–Meier and Cox proportional hazards analyses were performed using the SPSS version 17.0 (SPSS, Chicago, IL). For the genotype combination we used a statistical correction. Indeed, the *p* value for the statistical significance was obtained using 1000-fold permutation testing (software available on https://sourceforge.net/projects/mdr/files/mdrpt/), and the significance was set for values less than 0.05.

As an explorative study in nature, no estimation of power and sample size was performed because of the absence of previous published data regarding the specific investigated genetic profiles and the administered combination treatment. No data were excluded from the analysis.

### Reporting summary

Further information on research design is available in the Nature Research Reporting Summary linked to this article.

## Supplementary information

\lementary files

Reporting Summary

## Data Availability

The data that support the findings of this study are available from the corresponding author upon reasonable request.

## Code availability

The code for calculating HRs is written in SPSS version 17.0 and the code for identifying favorable/unfavorable profiles is written in Multifactor Dimensionality Reduction software version 2.0 beta 6. Codes are available upon request to M.S.
