# Supplementary material for: A pharmacogenetic interaction analysis of bevacizumab with paclitaxel in advanced breast cancer patients
Source: NPJ Breast Cancer. 2022 Mar 21;8:33. doi: 10.1038/s41523-022-00400-6 (PMC8938486; doi:10.1038/s41523-022-00400-6)
Supplement: Supplementary file 1 — \lementary files [file 41523_2022_400_MOESM1_ESM.pdf]

SUPPLEMENTARY FILES

A pharmacogenetic interaction analysis of bevacizumab with paclitaxel in advanced breast cancer patients

Luigi Coltelli°[1,2], Giacomo Allegrini°[1,2], Paola Orlandi°[3], Chiara Finale[1,2], Andrea Fontana[4], Luna Chiara Masini[1,2], Marco Scalese[5], Giada Arrighi[1,2], Maria Teresa Barletta[1,2], Ermelinda De Maio[1,2], Marta Banchi[3], Elisabetta Fini[3], Patrizia Guidi[3], Giada Frenzilli[3], Sara Donati[6], Simona Giovannelli[7], Lucia Tanganelli[7], Barbara Salvadori[4], Lorenzo Livi[8], Icro Meattini[8], Ilaria Pazzagli[9], Marco Di Lieto[9], Mirco Pistelli[10], Virginia Casadei[11], Antonella Ferro[12], Samanta Cupini[1,2], Francesca Orlandi[1,2], Damiana Francesca[1,13], Giulia Lorenzini[4], Leonardo Barellini[1,14], Alfredo Falcone[4,15], Alessandro Cosimi[1], Guido Bocci*[3].

[1]Department of Oncology, Azienda USL Toscana Nord Ovest, Italy; [2]Division of Medical Oncology, Livorno and Pontedera Hospitals, Azienda USL Toscana Nord Ovest, Italy; [3]Department of Clinical and Experimental Medicine, University of Pisa, Italy; [4]Division of Medical Oncology II, Azienda Ospedaliero-Universitaria Pisana, S. Chiara Hospital, Pisa, Italy; [5]Institute of Clinical Physiology, Italian National Research Council – CNR, Pisa Italy; [6]Division of Medical Oncology, Versilia Hospital, Azienda Usl Toscana Nord Ovest, Lido di Camaiore, Italy; [7]Division of Medical Oncology, San Luca Hospital, Azienda Usl Toscana Nord Ovest, Lucca, Italy; [8]Division of Radiotherapy, Azienda Ospedaliero-Universitaria Careggi, Firenze, Italy; [9]Division of Medical Oncology, Pescia and Pistoia Hospitals, Azienda Usl Toscana Centro, Pistoia, Italy; [10]Division of Medical Oncology, Umberto I Salesi-Lancisi Hospital, Azienda Ospedaliero-Universitaria Umberto I, Ancona, Italy; [11] Division of Medical Oncology, Marche Nord Hospital, Azienda Ospedaliera San Salvatore, Pesaro, Italy; [12]Division of Medical Oncology, Santa Chiara Hospital, Azienda Provinciale per I Servizi Sanitari, Trento, Italy; [13]Division of Radiology, Pontedera Hospital, Azienda Usl Toscana Nord Ovest, Pisa, Italy; [14]Breast Unit – Division of Breast Surgery, Livorno Hospital, Azienda Usl Toscana Nord Ovest, Livorno, Italy; [15]Department of Translational Research and New Technology in Medicine and Surgery, University of Pisa, Italy

° these authors equally contributed to the present work

*** Author for correspondence**

<u>Guido Bocci, MD PhD</u>
Department of Clinical and Experimental Medicine,
University of Pisa,
Via Roma 55, 56126 Pisa, Italy
e-mail: guido.bocci@unipi.it
phone: +390502218756

Supplementary table 1. Formal test of interaction confirming the predictive nature of the favorable profile in the bevacizumab + paclitaxel group. The interaction confirmed that the type of drugs (bevacizumab + paclitaxel vs. paclitaxel) significantly (p=0.007) modifies the effect that the genetic profile determines on PFS

Dependent variable PFS				
	Sig.	HR	CI Lower	CI Upper
Sites involvement (≥3 *vs*. <3)	0.042	1.328	1.010	1.747
Visceral disease (yes *vs.* no)	0.024	1.350	1.041	1.751
HR positive (yes *vs.* no)	0.003	0.608	0.438	0.844
Age years (<65 *vs*. >65)	0.011	0.702	0.535	0.923
Profile (unfavorable *vs*. favorable profile)	0.003	1.571	1.161	2.126
Drugs (paclitaxel *vs.* bevacizumab + paclitaxel)	0.017	1.875	1.120	3.137
**Profile*drugs (interaction)**	**0.007**	**0.452**	**0.253**	**0.808**

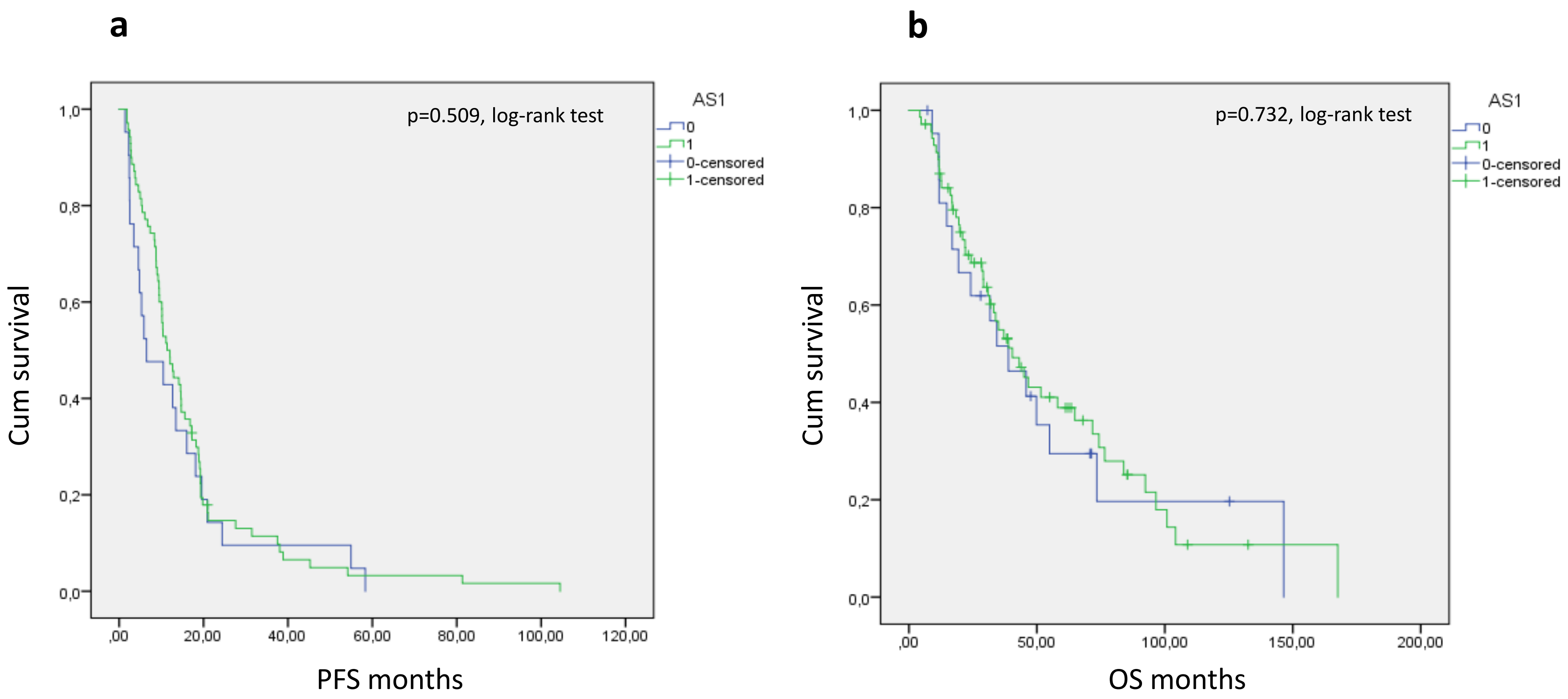

Supplementary figure 1. Progression-free survival (PFS; **a**) and overall survival (OS; **b**) curves in patients treated with paclitaxel calculated by the Kaplan Meier method, according to the favorable (blue line) and unfavorable (green line) genetic profiles of table 5.

Supplementary table 2. Results of the genetic interaction analysis to translate the genotype combinations of the *VEGFR-2* rs2071559 and *VEGFR-2* rs1870377 polymorphisms into favorable or unfavorable genetic profiles for progression free survival. *VEGFR-2, vascular endothelial growth factor receptor-2.*

Favorable genetic profiles			
VEGFR-2 rs2071559	AA	*VEGFR-2* rs1870377	TT
VEGFR-2 rs2071559	AA	*VEGFR-2* rs1870377	AT
VEGFR-2 rs2071559	AG	*VEGFR-2* rs1870377	AT
Unfavorable genetic profiles			
VEGFR-2 rs2071559	AA	*VEGFR-2* rs1870377	AA
VEGFR-2 rs2071559	AG	*VEGFR-2* rs1870377	TT
VEGFR-2 rs2071559	AG	*VEGFR-2* rs1870377	AA
VEGFR-2 rs2071559	GG	*VEGFR-2* rs1870377	TT
VEGFR-2 rs2071559	GG	*VEGFR-2* rs1870377	AT
VEGFR-2 rs2071559	GG	*VEGFR-2* rs1870377	AA

PFS	Median N° of months	N° of events
Favorable genetic profile	12.566 (95% CI: 10.441-14.69)	86
Unfavorable genetic profile	11.250 (95% CI: 9.982-12.518)	123

OS	Median N° of months	N° of events
Favorable genetic profile	32.599 (95% CI: 25.058-40.140)	65
Unfavorable genetic profile	27.368 (95% CI: 19.804-34.933)	105

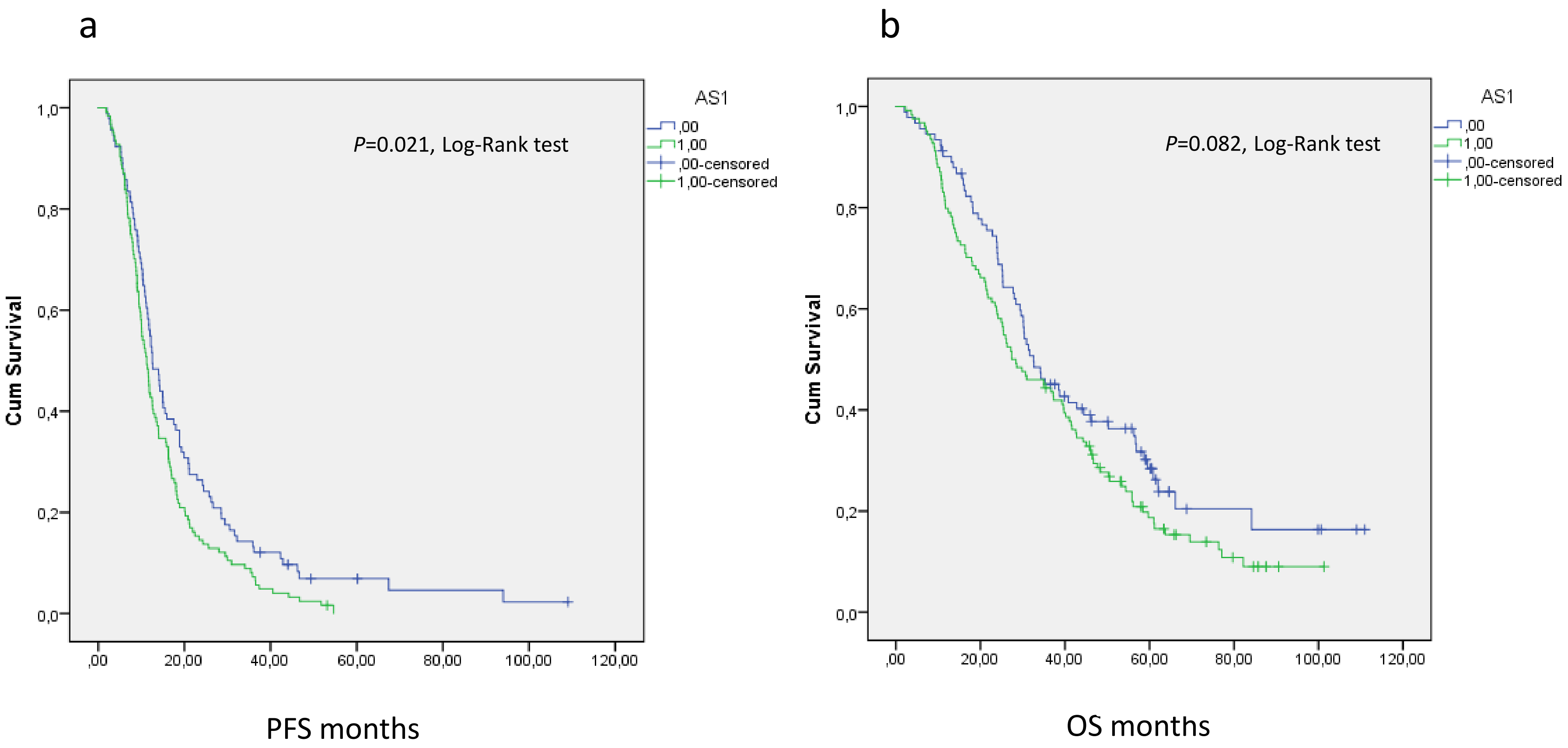

Supplementary figure 2. Progression-free survival (PFS; **a**) and Overall survival (OS; **b**) curves in patients treated with paclitaxel + bevacizumab calculated by the Kaplan Meier method, according to the favorable (blue line) and unfavorable (green line) genetic profiles of supplementary table 2.

Supplementary table 3. Genotype, allelic frequencies, and Hardy–Weinberg equilibrium of patient's population treated with bevacizumab and paclitaxel

Single Nucleotide Polymorphism	Genotype	Genotype frequency (%)	Allelic frequency (%)	Chi-square	Hardy-Weinberg equilibrium
VEGF-A	37AA	AA=17.2%	184A=42.8%	χ^2=0.43	P=0.51
rs699947	110AC	AC=51.2%			
	68CC	CC=31.6%	246C=57.2%		
VEGF-A	36CC	CC=16.7%	183C=42.6%	χ^2=0.67	P=0.41
rs833061	111CT	CT=51.7%			
	68TT	TT=31.6%	247T=57.4%		
VEGF-A	45GG	GG=20.9%	177G=41.2%	χ^2=5.83	P=0.02
rs1570360	87AG	AG=40.5%			
	83AA	AA=38.6%	253A=58.8%		
VEGF-A	82GG	GG=38.1%	273G=63.5%	χ^2=1.88	P=0.17
rs2010963	109GC	GC=50.7%			
	24CC	CC=11.2%	157C=36.5%		
VEGF-A	156CC	CC=72.6%	362C=84.2%	χ^2=3.45	P=0.06
rs3025039	50CT	CT=23.2%			
	9TT	TT=4.2%	68T=15.8%		
VEGF-A	123AA	AA=57.2%	329A=76.5%	χ^2=1.18	P=0.28
rs699946	83AG	AG=38.6%			
	9GG	GG=4.2%	101G=23.5%		
VEGFR-2	57AA	AA=26.5%	221A=51.4%	χ^2=0.003	P=0.95
rs2071559	107AG	AG=49.8%			
	51GG	GG=23.7%	209G=48.6%		

VEGFR-2	177CC	CC=82.3%	389C=90.5%	χ^2=0.68	P=0.41
rs2305948	35CT	CT=16.3%			
	3TT	TT=1.4%	41T=9.5%		
VEGFR-2	12AA	AA=5.6%	102A=23.7%	χ^2=0.001	P=0.97
rs1870377	78AT	AT=36.3%			
	125TT	TT=58.1%	328T=76.3%		
VEGFR-2	42CC	CC=19.5%	193C=44.9%	χ^2=0.13	P=0.72
rs11133360	109CT	CT=50.7%			
	64TT	TT=29.8%	237T=55.1%		
IL-8	39AA	AA=18.1%	192A=44.7%	χ^2=1.14	P=0.29
rs4073	114AT	AT=53.0%			
	62TT	TT=28.9%	238T=55.3%		
HIF-1α	44CC	CC=20.4%	252C=58.6%	χ^2=70.38	P=0.00
rs11549465	164CT	CT=76.3%			
	7TT	TT=3.3%	178T=41.4%		
EPAS-1	158GG	GG=73.5%	369G=85.8%	χ^2=0.03	P=0.85
rs4145836	53AG	AG=24.6%			
	4AA	AA=1.9%	61A=14.2%		

Supplementary table 4. Genotype, allelic frequencies, and Hardy–Weinberg equilibrium of patient's population treated with paclitaxel alone

Single Nucleotide Polymorphism	Genotype	Genotype frequency (%)	Allelic frequency (%)	Chi-square	Hardy-Weinberg equilibrium
VEGF-A	14AA	AA=15.2%	68A=37.0%	χ^2=0.41	P=0.52
rs699947	40AC	AC=43.5%			
	38CC	CC=41.3%	116C=63.0%		
VEGF-A	14CC	CC=15.2%	69C=37.5%	χ^2=0.22	P=0.64
rs833061	41CT	CT=44.6%			
	37TT	TT=40.2%	115T=62.5%		
VEGF-A	39GG	GG=42.4%	106G=57.6%	χ^2=13.07	P=0.0003
rs1570360	28AG	AG=30.4%			
	25AA	AA=27.2%	78A=42.4%		
VEGF-A	27GG	GG=29.3%	95G=51.6%	χ^2=1.07	P=0.30
rs2010963	41GC	GC=44.6%			
	24CC	CC=26.1%	89C=48.4%		
VEGF-A	71CC	CC=77.2%	162C=88.0%	χ^2=0.097	P=0.75
rs3025039	20CT	CT=21.7%			
	1TT	TT=1.1%	22T=12.0%		
VEGF-A	52AA	AA=56.5%	133A=72.3%	χ^2=4.19	P=0.04
rs699946	29AG	AG=31.5%			
	11GG	GG=12.0%	51G=27.7%		
VEGFR-2	34AA	AA=37.0%	108A=58.7%	χ^2=0.98	P=0.32
rs2071559	40AG	AG=43.5%			
	18GG	GG=19.5%	76G=41.3%		

VEGFR-2	72CC	CC=78.3%	162C=88.0%	χ^2=0.46	P=0.50
rs2305948	18CT	CT=19.6%			
	2TT	TT=2.1%	22T=12.0%		
VEGFR-2	14AA	AA=15.2%	55A=29.9%	χ^2=8.27	P=0.004
rs1870377	27AT	AT=29.4%			
	51TT	TT=55.4%	129T=70.1%		
VEGFR-2	23CC	CC=25.0%	78C=42.4%	χ^2=7.62	P= 0.006
rs11133360	32CT	CT=34.8%			
	37TT	TT=40.2%	106T=57.6%		
IL-8	17AA	AA=18.5%	80A=43.5%	χ^2=0.028	P=0.87
rs4073	46AT	AT=50.0%			
	29TT	TT=31.5%	104T=56.5%		
HIF-1α	42CC	CC=45.7%	130C=70.7%	χ^2=3.89	P=0.048
rs11549465	46CT	CT=50.0%			
	4TT	TT=4.3%	54T=29.3%		
EPAS-1	76GG	GG=82.6%	167G=90.8%	χ^2=0.071	P=0.79
rs4145836	15AG	AG=16.3%			
	1AA	AA=1.1%	17A=9.2%		
