# Supplementary material for: A pharmacogenetic interaction analysis of bevacizumab with paclitaxel in advanced breast cancer patients
Source: NPJ Breast Cancer. 2022 Mar 21;8:33. doi: 10.1038/s41523-022-00400-6 (PMC8938486; doi:10.1038/s41523-022-00400-6)
Supplement: Supplementary file 2 — Reporting Summary [file 41523_2022_400_MOESM2_ESM.pdf]

nature portfolio

Corresponding author(s): DBPR NPJBCANCER-01431

Last updated by author(s): Nov 24, 2021

Reporting Summary

Nature Portfolio wishes to improve the reproducibility of the work that we publish. This form provides structure for consistency and transparency in reporting. For further information on Nature Portfolio policies, see our Editorial Policies and the Editorial Policy Checklist.

Statistics

For all statistical analyses, confirm that the following items are present in the figure legend, table legend, main text, or Methods section.

n/a	Confirmed	
☐	☒	The exact sample size (*n*) for each experimental group/condition, given as a discrete number and unit of measurement
☐	☒	A statement on whether measurements were taken from distinct samples or whether the same sample was measured repeatedly
☐	☒	The statistical test(s) used AND whether they are one- or two-sided<br>*Only common tests should be described solely by name; describe more complex techniques in the Methods section.*
☐	☒	A description of all covariates tested
☐	☒	A description of any assumptions or corrections, such as tests of normality and adjustment for multiple comparisons
☐	☒	A full description of the statistical parameters including central tendency (e.g. means) or other basic estimates (e.g. regression coefficient) AND variation (e.g. standard deviation) or associated estimates of uncertainty (e.g. confidence intervals)
☐	☒	For null hypothesis testing, the test statistic (e.g. *F*, *t*, *r*) with confidence intervals, effect sizes, degrees of freedom and *P* value noted<br>*Give P values as exact values whenever suitable.*
☒	☐	For Bayesian analysis, information on the choice of priors and Markov chain Monte Carlo settings
☒	☐	For hierarchical and complex designs, identification of the appropriate level for tests and full reporting of outcomes
☐	☒	Estimates of effect sizes (e.g. Cohen's *d*, Pearson's *r*), indicating how they were calculated

Our web collection on statistics for biologists contains articles on many of the points above.

Software and code

Policy information about availability of computer code

Data collection	software Excel, microsoft office.
Data analysis	MDR software version 2.0 beta 6 on http://sourceforge.net/projects/mdr/<br>SPSS version 17.0 (SPSS, Chicago, IL)

For manuscripts utilizing custom algorithms or software that are central to the research but not yet described in published literature, software must be made available to editors and reviewers. We strongly encourage code deposition in a community repository (e.g. GitHub). See the Nature Portfolio guidelines for submitting code & software for further information.

Data

Policy information about availability of data

All manuscripts must include a data availability statement. This statement should provide the following information, where applicable:

- Accession codes, unique identifiers, or web links for publicly available datasets
- A description of any restrictions on data availability
- For clinical datasets or third party data, please ensure that the statement adheres to our policy

The data that support the findings of this study are available from the corresponding author upon reasonable request

Field-specific reporting

Please select the one below that is the best fit for your research. If you are not sure, read the appropriate sections before making your selection.

☒ Life sciences ☐ Behavioural & social sciences ☐ Ecological, evolutionary & environmental sciences

For a reference copy of the document with all sections, see nature.com/documents/nr-reporting-summary-flat.pdf

Life sciences study design

All studies must disclose on these points even when the disclosure is negative.

Sample size	As an explorative study in nature, no estimation of power and sample size was performed because of the absence of previous published data regarding the specific investigated genetic profiles and the administered combination treatment. The same exploratory approach has been previously published by Pander and colleagues (Pander, J., Wessels, J. A. M., Gelderblom, H., van der Straaten, T., Punt, C. J. A. & Guchelaar, H. J. Pharmacogenetic interaction analysis for the efficacy of systemic treatment in metastatic colorectal cancer. Ann. Oncol. 22, 1147–1153 (2011).
Data exclusions	No data were excluded from the analysis
Replication	All the samples were analyzed twice with validated TaqMan® SNP genotyping assays to replicate the obtained genotype.
Randomization	The participants were retrospectively allocated into the two groups depending on the administered therapy: paclitaxel plus bevacizumab vs. paclitaxel alone. No randomization was performed but no statistical differences were found for the baseline characteristics of the enrolled patients (see table 1)
Blinding	The investigators responsible for data analysis were blinded to which samples were from patients treated with paclitaxel alone and paclitaxel plus bevacizumab.

Reporting for specific materials, systems and methods

We require information from authors about some types of materials, experimental systems and methods used in many studies. Here, indicate whether each material, system or method listed is relevant to your study. If you are not sure if a list item applies to your research, read the appropriate section before selecting a response.

Materials & experimental systems

n/a	Involved in the study	
☒	☐	Antibodies
☒	☐	Eukaryotic cell lines
☒	☐	Palaeontology and archaeology
☒	☐	Animals and other organisms
☐	☒	Human research participants
☐	☒	Clinical data
☒	☐	Dual use research of concern

Methods

n/a	Involved in the study	
☒	☐	ChIP-seq
☒	☐	Flow cytometry
☒	☐	MRI-based neuroimaging

Human research participants

Policy information about studies involving human research participants

Population characteristics	Basal and pathological characteristics recorded from both groups were the following: age (≤ or >65 years); Eastern Cooperative Oncology Group (ECOG) performance status (0 or 1-2); hormonal-receptor status (positive or negative); previous adjuvant chemotherapy (none, anthracycline or anthracycline plus taxanes); previous hormonal therapy (adjuvant or metastatic); disease-free interval from the first diagnosis of breast cancer (≤ or >12 months); extent of disease (≤ or >3 sites); location of disease (viscera or bone); disease evaluation (measurable or non-measurable). Patients with human epidermal growth factor receptor type 2 (HER2)-positive, were excluded from the present study. See table 1
Recruitment	This is an explorative, ambidirectional cohort study, meaning that eligible patients were enrolled and evaluated retrospectively from January 2009 until September 2016 and then followed prospectively. The oncology units, all located in the north or centre of Italy, were selected based on their clinical experience in the use of the combination of paclitaxel and bevacizumab as first-line therapy in histologically confirmed HER-2-negative MBC patients. Ambidirectional studies are much less common than purely prospective or retrospective studies, but they are conceptually consistent with and share elements of the advantages and disadvantages of both types of studies.
Ethics oversight	The protocol was approved by ethic committee of Azienda Ospedaliera-Universitaria Pisana (CESM-AOUP 3077/2010; clinicaltrials.gov identifier NCT01935102)

Note that full information on the approval of the study protocol must also be provided in the manuscript.

Clinical data

Policy information about clinical studies

All manuscripts should comply with the ICMJE guidelines for publication of clinical research and a completed CONSORT checklist must be included with all submissions.

Clinical trial registration	clinicaltrials.gov identifier NCT01935102
Study protocol	the full protocol is available at the ethic committee of Azienda Ospedaliera-Universitaria Pisana (CESM-AOUP), Pisa, Italy
Data collection	the recrutiment and data collection were from January 2009 until september 2016 in 11 italian oncology units. The genotyping analysis was not performed until an adequate number of events (>80% on study population) was reported in terms of PFS at the Unit of Pharmacology of the School of Medicine of the University of Pisa
Outcomes	PFS was defined as the period from the beginning of the treatment to the first observation of disease progression as above described, or death from any cause. OS was defined as the period from the beginning of the treatment to death from any cause.
